# Epigenetic Perspectives and Their Prognostic Value in Early Recurrence After Hepatocellular Carcinoma Resection

**DOI:** 10.3390/cancers17050769

**Published:** 2025-02-24

**Authors:** Chang-Yi Lu, Ching-Pin Lin, Hsiang-Lin Lee, Pey-Jey Peng, Shao-Chang Huang, Meng-Rong Chuang, Yih-Jyh Lin

**Affiliations:** 1 Department of Clinical Diagnostic Technology, EpiSante Biomedical Co., Ltd., Hsinchu 302, Taiwan; cylu@episante-biomed.com (C.-Y.L.);; 2 Department of Internal Medicine, Chung Shan Medical University Hospital, Taichung 402, Taiwan; 3 School of Medicine, Chung Shan Medical University, Taichung 402, Taiwan; 4 Department of Surgery, Chung Shan Medical University Hospital, Taichung 402, Taiwan; 5 Division of General and Transplant Surgery, Department of Surgery, National Cheng Kung University Hospital, College of Medicine, National Cheng Kung University, Tainan 704, Taiwan; 6 Liver Cancer Collaborative Oncology Group, National Cheng Kung University Hospital, Tainan 704, Taiwan

**Keywords:** hepatocellular carcinoma, early recurrence, DNA methylation

## Abstract

Background/Objectives: The post-hepatectomy survival of patients with hepatocellular carcinoma (HCC) faces challenges due to high recurrence rates, especially early recurrence (ER). We investigated DNA methylation in HCC and developed a methylation-based model for ER prediction (MER). Methods: We studied HCC patients with ER within a year post-hepatectomy, comparing them to those who remained recurrence-free (RF) for 5 years. In a testing set, we examined genome-wide methylation profiles to identify differences between ER and RF. Validation in an independent cohort confirmed candidate markers using real-time quantitative methylation-specific PCR (qMSP). We constructed MER by incorporating identified gene methylation, clinical information, and serum protein marker, and evaluated its predictive performance using ROC analysis and Cox regression. Results: Distinct signatures of hypermethylation and hypomethylation were observed between ER and RF, as well as between cirrhotic and non-cirrhotic groups. Significant aberrant methylation pathways, including FGFR signaling, the PI3K network, and the MAPK pathway, were observed in non-cirrhotic ER patients. Conversely, cirrhotic ER patients showed notable associations with Wnt/β-catenin signaling, cell adhesion, and migration mechanisms. Through qMSP analysis, we identified ER-associated genes, including BDNF, FOXL2, LMO7, NCAM1, NEIS3, PLA2G7, and LTB4R. MER demonstrated strong predictive ability for ER, with an AUC of 0.855, surpassing current indicators such as AFP, tumor size, and BCLC stage. Combining different predictors resulted in heightened AUC values. Importantly, the inclusion of MER yielded to the highest AUC of 0.952, underscoring the substantial contribution of MER to predictive accuracy. Conclusions: This study discovered the involvement of aberrant DNA methylation in HCC with early recurrence. The MER outperforms clinicopathological predictors and achieves robust prediction capabilities in identifying patients at risk of ER.

## Simple Summary

This study explored the role of DNA methylation in the early recurrence (ER) of hepatocellular carcinoma (HCC) after resection. By analyzing genome-wide methylation patterns, distinct signatures associated with ER and the involvement of aberrant methylation in crucial signaling pathways were identified. A novel methylation-based model (MER) was developed, demonstrating superior predictive accuracy for ER compared to traditional clinical predictors. This model holds promises for improved risk stratification and personalized treatment strategies for HCC patients.

## 1. Introduction

Hepatocellular carcinoma (HCC) is a highly lethal form of cancer that poses a significant burden on the healthcare system due to its widespread occurrence. Various treatment approaches have been proposed and employed based on staging systems. Among them, hepatic resection is commonly chosen as the primary treatment for patients with early-stage HCC, as it offers the potential for a cure. Nevertheless, the long-term effectiveness of hepatectomy remains unsatisfactory due to a high recurrence rate, which has been reported to reach 70% within five years after the operation [1].

The recurrence of HCC after curative hepatic resection follows a bimodal pattern, characterized by early and late recurrence [2]. This pattern sheds light on the nature of tumor recurrence after hepatectomy and underscores the need for adjuvant therapy. Early recurrence is typically associated with poor prognosis. Therefore, the early detection or prediction of early recurrence before it occurs is of utmost importance.

In response to the pressing need for early recurrence prediction, numerous predictive protocols have been proposed. These protocols consist of clinicopathological features, biomarkers, gene-related signatures, or their combinations. Despite their limited effectiveness, criteria that utilize clinicopathological features continue to be extensively employed for risk stratification in trial design and clinical practices.

Among gene-related signatures in cancer study, DNA methylation is one of the most important epigenetic regulations and plays a fundamental role in every stage of carcinogenesis by regulating cancer-related gene expression. Increasing evidence shows that DNA hypermethylation is involved in the mechanism of repressing tumor suppressors and DNA repair genes, and DNA hypomethylation is responsible for the overexpression of oncogenes in many types of cancer, including HCC [3]. The use of these aberrantly methylated genes as cancer markers is emerging for the value of clinical applications in diagnosis, prognosis, therapy selection, and recurrence monitoring [4]. In this study, we try to address the critical issue of early recurrence after the surgical resection of liver cancer from an epigenetic perspective. At first, we analyzed genome-wide methylation profiles to identify differential methylation changes between HCC patients with and without early recurrence and delineate the distinct pathways that are involved. Potential methylation markers were further validated by real-time quantitative methylation-specific PCR (qMSP), and a prediction model was established to accurately distinguish early recurrence in HCC patients after hepatectomy.

## 2. Materials and Methods

### 2.1. Selection of Patients and Clinical Samples

A total of 64 HCC patients undergoing surgical resection (24 patients for testing set and 40 patients for validation set) were enrolled from National Cheng Kung University (NCKU) Hospital, Taiwan. We defined patients who experienced a relapse within one year after liver resection as “early recurrence” (ER) patients, whereas those who remain recurrence-free for over five years as “recurrence free” (RF) patients (Table 1). All patients included in the study were diagnosed with incident HCC and received hepatic resection as their initial treatment. Tumor tissue samples were promptly collected from surgically excised specimens in the operating room, immediately frozen using liquid nitrogen, and stored at −80 °C until required. The sample collection and storage procedures were conducted at NCKUH biobank. We randomly selected only those patients who met the ER or RF criteria and had samples with sufficient volume and optimal storage conditions. The Institutional Review Board of Human Research of NCKU Hospital approved all experimental protocols and study methods. Written informed consent was obtained from the patients who provided the specimens.

Genomic DNA was extracted from tumor tissue by using the DNeasy blood and tissue kit (Qiagen, Redwood, CA, USA). The quality and quantity of the isolated genomic DNA were analyzed with an ND-1000 spectrophotometer (Thermo, Waltham, MA, USA).

### 2.2. DNA Methylation Microarray and Functional Analysis

Infinium Human Methylation 450K BeadChip (Illumina, San Diego, CA, USA) was used to identify novel methylated genes involved in early recurrence after resection. A total of 500 ng of DNA was bisulfite converted using the EZ DNA Methylation Kit (Zymo Research, Irvine, CA, USA) according to the manufacturer’s protocol and processed on the bead array. The methylation level for each probe was calculated as an average beta value scaled from 0 (unmethylated) to 1 (completely methylated), representing the ratio of methylation at a specific CpG locus. To identify differentially methylated regions, array data were processed using several filtering steps, consisting of removing CpG sites with a detection *p*-value ≥ 0.05, with beadcount < 3 on the X and Y chromosomes and containing documented single nucleotide polymorphisms (SNPs).

To explore the potential mechanisms of the genes affected by DNA methylation, significantly differential methylated genes were subjected to Gene Ontology (GO) enrichment (http://geneontology.org/, accessed on 22 September 2022) and Kyoto Encyclopedia of Genes and Genomes (KEGG) pathway analyses (https://www.kegg.jp/, accessed on 3 October 2022) using the Database for Annotation, Visualization, and Integrated Discovery (DAVID), which is a comprehensive set of functional annotation tools (https://david.ncifcrf.gov/, accessed on 4 October 2022). GO terms include biological process, cellular component, and molecular function. The specific cut-off used for terms and pathways was 0.05, and the top 10 GO terms and pathways were selected.

The interaction network of differentially methylated genes was constructed using the search tool for the retrieval of interacting genes (STRING) (https://string-db.org, accessed on 6 December 2022) database, which integrates both known and predicted protein–protein interactions (PPIs). In the networks, the nodes correspond to the proteins and the edges represent the interactions. The interactions include direct (physical) and indirect (functional) associations. Interaction sources included experimental repositories, computational prediction methods, and public text collections. The selected species was limited to Homo sapiens, and a combined score  >  0.4 was applied. For the network, the PPI enrichment *p*-value and average local clustering coefficient were reported.

### 2.3. Combined Bisulfite Restriction Analysis (COBRA)

One microgram of genomic DNA from HCC clinical samples was bisulfite-converted using the EZ DNA Methylation Kit (Zymo Research). The converted DNA was then amplified by PCR using the Kapa SYBR Fast qPCR Kit (Kapa Biosystems, Wilmington, MA, USA). Each 20 μL PCR reaction contained 1 μL of converted DNA, 0.5 μM of each primer, and 1× Kapa SYBR Fast qPCR Master Mix. The PCR cycling conditions were 95 °C for 3 min; 40 cycles of 95 °C for 3 s; an annealing temperature for 20 s; and 72 °C for 10 s; followed by a final extension at 72 °C for 20 s. Primer and probe sequences are available upon request. The amplified DNA was digested with restriction enzymes targeting CpG sites within their recognition sequences. The resulting fragments were visualized on a 1.5% (*w*/*v*) ethidium bromide-stained agarose gel.

### 2.4. Real-Time Quantitative Methylation Analysis

The bisulfite-converted DNA was amplified using real-time quantitative methylation-specific PCR (qMSP) with fluorescent probes. Each 20 μL reaction contained 1× Kapa Probe Fast qPCR Master Mix, 0.5 μM of each primer, and 0.25 μM of probe. The amplification process was carried out on a StepOnePlus Real-Time PCR System (Thermo Fisher Scientific, Waltham, MA, USA). Primer and probe sequences can be provided upon request. Methylation levels were calculated using the previously described formula as follows: 2^[Ct(beta-actin)−Ct(candidate)]×100^.

### 2.5. Statistical Analysis

An independent samples *t*-test was used to determine the difference in methylation levels in ER and RF patients. The logistic regression models were used to establish a “methylation prediction model for ER” (MER). Receiver operating characteristic (ROC) curve analysis was performed to assess diagnostic performance, estimating the area under the curve (AUC), cutoff value, sensitivity, and specificity. Cox regression analysis was used to evaluate the association between each variable and survival. Kaplan–Meier methods were used to generate survival curves, and the log-rank test was used to compare distributions. Disease-free survival was defined as the time from diagnosis to recurrence or the end of follow-up, while overall survival was defined as the time from diagnosis to disease-related death or the end of follow-up. Multivariate analyses were performed using a Cox proportional hazards model to estimate hazard ratios (HRs) and their 95% confidence intervals (CIs). Statistical significance was defined as *p* < 0.05 for all tests.

## 3. Results

### 3.1. Identification of Differential Methylation Regions in Early Recurrent HCC Patients

**Figure 1:**
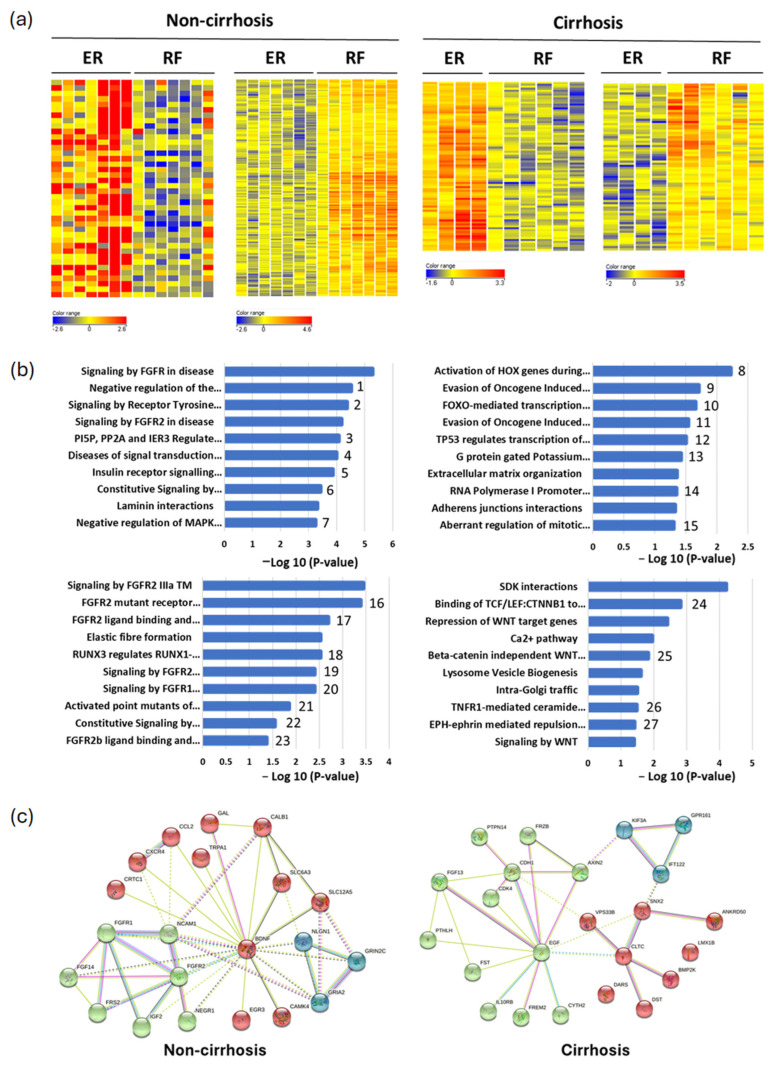
Differentially methylated genes in HCC early recurrence. (**a**) Unsupervised hierarchical cluster analysis of data from CpG microarrays in 24 HCC patients composed of 14 non-cirrhotic patients and 10 cirrhotic patients. Each row represents a gene, and each column represents a tissue sample. Hypermethylated and hypomethylated genes are indicated by red and blue, respectively. (**b**) Gene ontology and pathway analysis of aberrantly methylated genes. All pathways presented are statistically significant (*p* < 0.05). Note: 1. negative regulation of MAPK pathway; 2. signaling by receptor tyrosine kinases; 3. PI5P, PP2A and IER3 regulate PI3K/AKT signaling; 4. diseases of signal transduction by growth factor receptors and second messengers; 5. insulin receptor signalling cascade; 6. constitutive signaling by aberrant PI3K in cancer; 7. negative regulation of MAPK pathway; 8. activation of HOX genes during differentiation; 9. evasion of oncogene induced senescence due to defective p16INK4A binding to CDK4; 10. FOXO-mediated transcription of cell death genes; 11. evasion of oncogene induced senescence due to defective p16INK4A binding to CDK4; 12. TP53 regulates transcription of several additional cell death genes whose specific roles in p53-dependent apoptosis remain uncertain; 13. G protein gated potassium channels; 14. RNA polymerase I promoter opening; 15. aberrant regulation of mitotic cell cycle due to RB1 defects; 16. FGFR2 mutant receptor activation; 17. FGFR2 ligand binding and activation; 18. RUNX3 regulates RUNX1-mediated transcription; 19. signaling by FGFR2 amplification mutants; 20. signaling by FGFR1 amplification mutants; 21. activated point mutants of FGFR2; 22. constitutive signaling by aberrant PI3K in cancer; 23. FGFR2b ligand binding and activation; 24. binding of TCF/LEF:CTNNB1 to target gene promoters; 25. beta-catenin independent WNT signaling; 26. TNFR1-mediated ceramide production; 27. EPH-ephrin mediated repulsion of cells (**c**) Interaction networks of differentially methylated genes related to early recurrence in non-cirrhotic and cirrhotic HCCs. The network of non-cirrhotic HCC consists of 21 nodes and 45 edges, with an enrichment *p*-value < 1.0 × 10^−16^. The network of cirrhotic HCC consists of 23 nodes and 24 edges, with an enrichment *p*-value of 4.47 × 10^−11^. Each node represents all the proteins produced by a single protein coding gene locus and each edge represents the predicted functional associations.

To identify novel potential methylation markers, a genome-wide approach was used for this study in the first 24 enrolled patients, with 11 ER patients and 13 RF patients (Table 1). Through unsupervised hierarchical clustering, we observed distinct signatures of hypermethylation and hypomethylation between the ER and RF groups, with further variations seen between cirrhotic and non-cirrhotic patients (Figure 1a). In the non-cirrhotic subgroup, 40 significant hypermethylated regions and 297 significant hypomethylated regions of the ER group were identified. In the cirrhotic subgroup, 181 hypermethylated and 77 hypomethylated regions were obtained in the ER group (Figure 1a).

To further explore the mechanisms involved in these aberrantly methylated genes, gene ontology and pathway analysis were conducted. Interestingly, both hypermethylated and hypomethylated genes in the ER group are involved in FGFR signaling and the PI3K network in non-cirrhotic HCC patients (Figure 1b). Several important molecular changes in cellular adhesion, differentiation, and migration were observed, such as elastic fiber formation, RUNX3/RUNX1-mediated transcription regulation, and laminin interaction. Alterations in the MAPK pathway, insulin receptor, and tyrosine kinase signaling were also observed in the hypomethylated ER group of non-cirrhotic HCC patients. Conversely, cirrhotic patients exhibit markedly distinct pathways. In cirrhotic HCC, the ER group with hypermethylation exhibits the top 10 prominent pathways, which encompass the activation of HOX genes, defective p16INK4A binding to CDK4 and CDK6, FOXO-mediated transcription of cell death genes, TP53 regulated transcription, G protein-gated potassium channels, extracellular matrix organization, adherent junction interactions, and the aberrant regulation of the mitotic cell cycle due to RB1 defects. On the contrary, in cirrhotic patients belonging to the hypomethylated ER group, the most affected pathways involve Wnt/beta-catenin signaling and various cell adhesion and migration mechanisms, including SDK (Sidekick-1 and Sidekick-2) interactions and Eph/ephrin signaling (Figure 1b). These findings indicate considerable differences in the regulation of DNA methylation during the carcinogenesis of HCC between patients with cirrhosis and those without. Overall, HCC patients without cirrhosis typically display aberrant FGFR, PI3K/AKT, and MAPK signaling. By contrast, impaired cell cycle regulators and the Wnt pathway are observed in HCC patients with cirrhosis. Reasonably, several mechanisms of cell adhesion and migration are involved in the early recurrence of both cirrhotic and non-cirrhotic HCCs.

To build up the direct interaction networks of genes regulated by DNA methylation and identify key hub genes, 337 differentially methylated regions (40 hypermethylated regions and 297 hypomethylated regions) of non-cirrhotic HCC patients and 258 differentially methylated regions (181 hypermethylated regions and 77 hypomethylated regions) of HCCs with cirrhosis were separately analyzed by the Search Tool for Retrieval of Interacting Genes/Proteins (STRING). The enrichment and clustering showed several hub genes in differentially methylated regions of non-cirrhotic HCCs, including BDNF, FGFR1, and FGFR2. In differentially methylated regions of cirrhotic HCCs, EGF is dominated by STRING analysis (Figure 1c). These results correspond to previous findings and strongly indicate that EGFR and Wnt signaling crosstalk transactivates one another in cancer development.

### 3.2. Validation of Differential Methylated Genes by COBRA (Combined Bisulfite Restriction Analysis)

**Figure 2:**
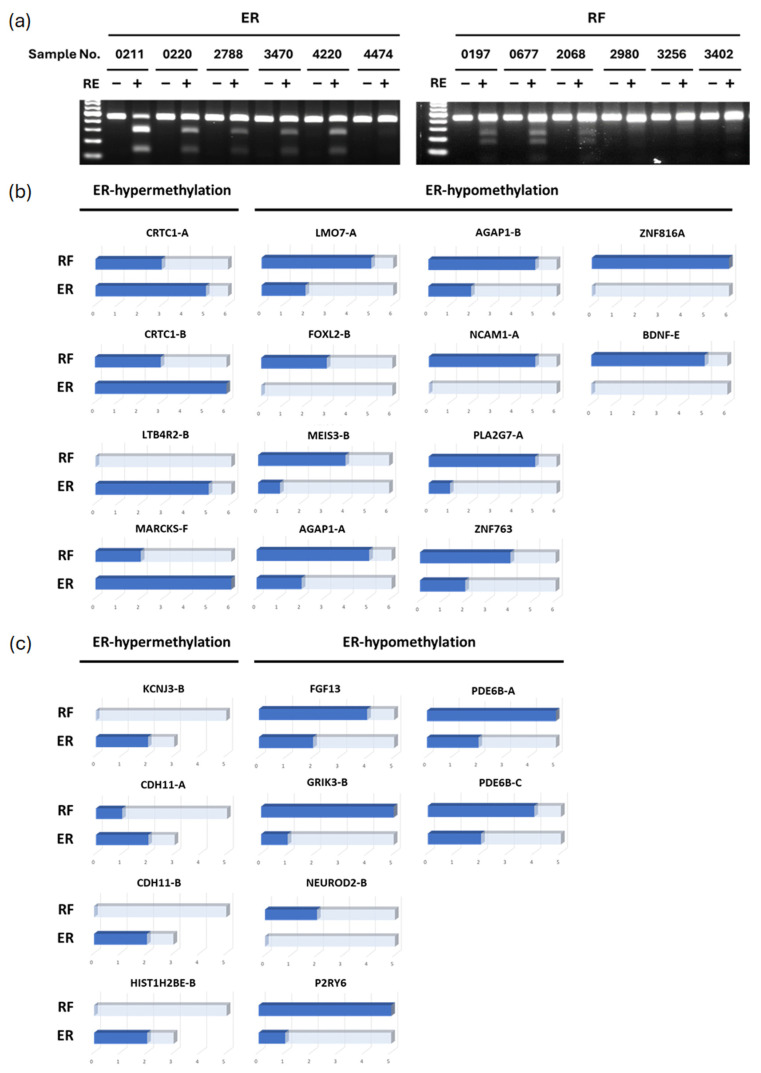
COBRA of differentially methylated genes in HCC patients. (**a**) The representative COBRA result of CRTC1 in 6 ER and 6 RF non-cirrhotic HCC patients. Following PCR amplification of sodium bisulfite-converted DNA, PCR products were incubated with or without a restriction enzyme (RE) as indicated by the plus or minus sign. The first lane is a 100 bp marker. (**b**) The summary of COBRA results in non-cirrhotic HCC patients composed of 6 ER and 6 RF patients. The dark blue bar represents the case showing hypermethylation. A single gene may have multiple COBRA PCR regions designed, which are denoted by an alphabet following the gene name. (**c**) The summary of COBRA results in cirrhotic HCC patients. Five ER patients and 5 RF patients were tested for hypomethylated genes, and 3 ER patients and 5 RF patients were tested for hypermethylated genes.

We used COBRA, a method of qualitative methylation assay, to validate the results obtained from the Human Methylation Array. In total, 22 clinical samples were examined by COBRA, including 12 non-cirrhotic patients and 10 cirrhotic patients. Figure 2a shows a representative example of the COBRA result for CRTC1. CRTC1 hypermethylation can be observed in five out of six ER patients with non-cirrhotic HCCs. By contrast, only three RF patients show CRTC1 hypermethylation. In non-cirrhotic HCC patients, CRTC1, LTB4R2, and MARCKS show hypermethylation in ER patients. Conversely, LMO7, FOXL2, MEIS3, AGAP1, BDNF, NCAM1, PLA2G7, ZNF763, and ZNF816A are found to be hypomethylated in ER patients (Figure 2b). In cirrhotic patients, KCNJ3, CDH11, and HIST1H2BE are hypermethylated, while FGF13, GRIK3, NEUROD2, P2RY6, and PDE6B are hypomethylated in ER patients compared to RF patients (Figure 2c).

### 3.3. Methylation Levels of Candidate Markers in Clinical Samples

**Figure 3:**
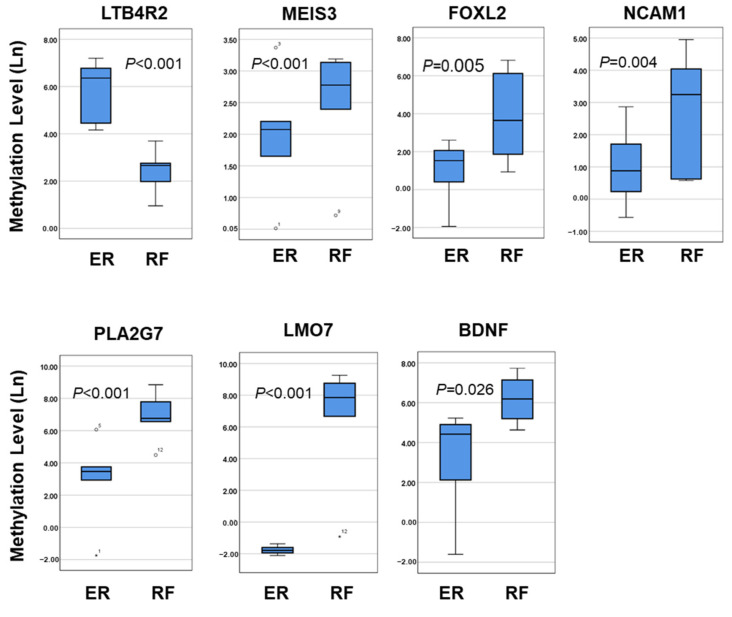
Methylation levels of candidate genes in ER and RF patients. Methylation levels of 7 candidate markers, including 1 hypermethylated gene and 6 hypomethylated genes, were determined by qMSP in ER and RF patients. Methylation levels were transformed by nature log and are depicted by box plots. Boxes extend from the 25th to 75th percentiles and are divided by a solid line that represents the median of each group. Whiskers extend from the 5th to the 95th percentiles. Each outlier is denoted by a dot. The *t*-test was used to determine statistical significance, except for BDNF, which was tested using the Mann–Whitney U test.

Previous studies have found that cirrhosis is a major risk factor for late recurrence after liver resection [5,6]. Firstly, we focused on the predicted methylation markers in non-cirrhotic HCC patients and recruited an additional 40 non-cirrhotic HCCs as a validation set, comprising 20 patients each from the ER and RF patients. According to the COBRA results, we designed both hypermethylated and hypomethylated candidate genes for qMSP testing. There are seven genes that showed significant disparities in methylation levels between ER and RF patients. LTB4R2 exhibits hypermethylation, while MEIS3, FOXL2, PLA2G7, LMO7, BDNF, and NCAM1 show hypomethylation in ER patients (Figure 3). The qMSP results coincide with those of COBRA, and these genes may potentially act as methylation markers for ER prediction.

### 3.4. Performance of Methylation Prediction Model for ER Prediction

**Figure 4:**
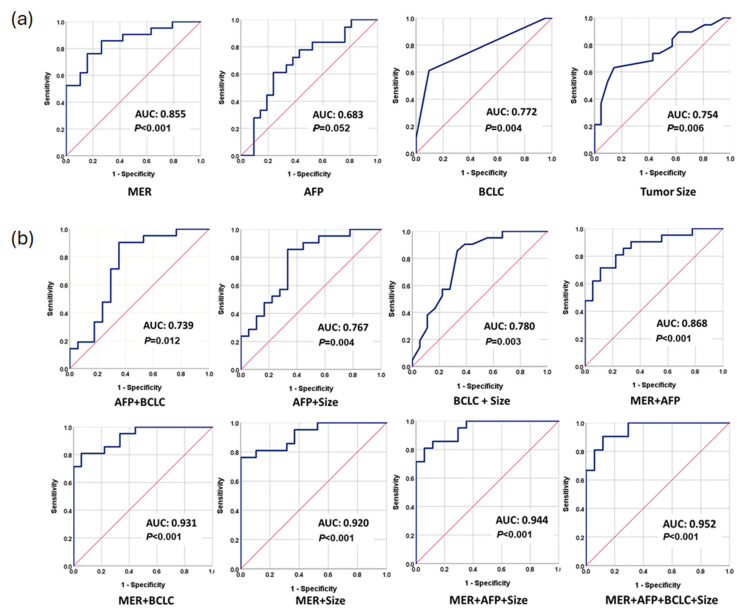
Receiver operator characteristic (ROC) curves for discriminating ER and RF patients. (**a**) ROC analysis for MER, AFP, BCLC, and tumor size, separately. (**b**) ROC analysis for the combination of MER, AFP, BCLC, and tumor size for each other and all of them as indicated. The red line is a reference line.

Logistic regression analysis was used to generate a prediction model with differentially methylated genes as follows: MER = −2.126 − 0.102 × ln(methylation level of LMO7) + 0.328 × ln(methylation level of NCAM1) − 0.373 × ln(methylation level of BDNF) −0.026 × ln(methylation level of MEIS3) + 0.066 × ln(methylation level of LTB4R2) − 0.047 × ln(methylation level of PLA2G7) − 0.370 × ln(methylation level of FOXL2). The performance of methylation prediction model for ER (MER) was assessed by receiver operating characteristic (ROC) analysis. The area under the curve (AUC) of MER was 0.855 (95% CI: 0.738–0.971, *p* < 0.001) (Figure 4a). The AUCs of AFP, BCLC, and tumor size alone were 0.683 (95% CI: 0.511–0.854, *p* = 0.052), 0.772 (95% CI: 0.619–0.926, *p* = 0.004), and 0.754 (95% CI: 0.600–0.909, *p* = 0.006), respectively (Figure 4a). By combining any two current predictors, there was a notable increase in the AUC, indicating an enhancement in predictive capacity. The AUC values ranged from 0.739 (95% CI: 0.569–0.910, *p* = 0.012) for AFP and BCLC to 0.780 (95% CI: 0.629–0.932, *p* = 0.003) for tumor size and BCLC (Figure 4b). Moreover, integrating MER with AFP, BCLC, or tumor size led to a substantial elevation in the AUCs, reaching 0.868 (95% CI: 0.756–0.979, *p* < 0.001), 0.931 (95% CI: 0.857–1.000, *p* < 0.001), or 0.920 (95% CI: 0.838–1.000, *p* < 0.001), respectively. The combination of all three current predictors and MER yielded the highest AUC value of 0.952 (95% CI: 0.893–1.000, *p* < 0.001) (Figure 4b, Table 2), underscoring a significant improvement in predictive performance.

The ROC analysis revealed distinct sensitivities and specificities for each predictor. Among them, MER showed a modest yet balanced performance with a sensitivity of 85.7% and specificity of 73.7% (Table 2). In comparison, tumor size had a sensitivity of 73.7% and specificity of 57.1%, AFP demonstrated a sensitivity of 72.2% and specificity of 61.9%, while BCLC displayed a sensitivity of 61.1% and an impressive specificity of 90.5%. Combining MER with AFP, tumor size, and BCLC resulted in improved sensitivity and specificity values of 85.7%/72.7%, 81.0%/89.5%, and 81.0%/94.4%, respectively (Table 2). Most remarkably, the combination of all three clinical predictors with MER resulted in an outstanding sensitivity of 90.5% and specificity of 88.2%. These findings unequivocally highlight the superior performance of MER over individual predictors or any combination thereof. MER, integrated with all clinical predictors, plays a pivotal role in providing a highly precise predictive ability for early recurrence in HCC patients after hepatectomy.

### 3.5. Methylation Prediction Model in Predicting Survival

**Figure 5:**
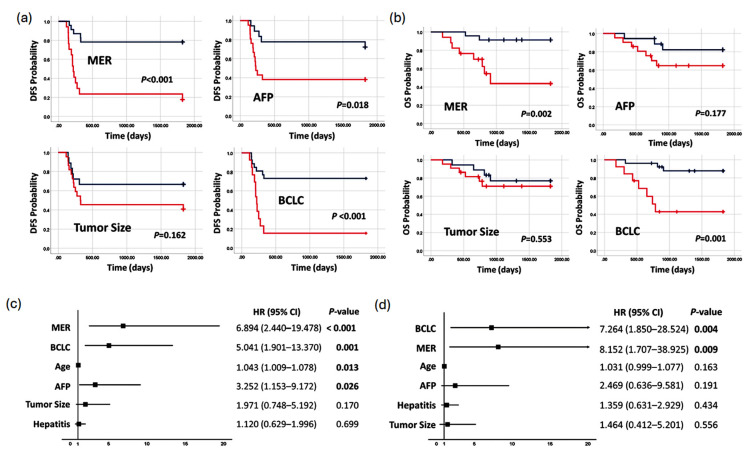
The prognostic abilities of predictors. Kaplan–Meier analysis of (**a**) disease-free survival (DFS) and (**b**) overall survival (OS) for HCC patients is classified into high- and low-risk groups according to MER with a cutoff value of 0.45, indicated by the red line and blue line, respectively. In the case of AFP, AFP ≥ 20 ng/mL is shown by the red line, and AFP < 20 ng/mL is shown by the blue line. For tumor size, the red line represents tumors with a diameter ≥ 5 cm, and the blue line represents tumors with a diameter < 5 cm. In terms of BCLC staging, stages 0 and A are shown by the blue line, whereas stages B and C are shown by the red line. Forest plot showing hazard ratios for (**c**) disease-free survival (DFS) and (**d**) overall survival (OS). The forest plots indicate the hazard ratios (HRs) and 95% confidence intervals (CIs) according to each risk factor that was estimated by a Cox proportional hazards model. *p* values < 0.05 are noted in bold.

Furthermore, we proceeded to assess its predicting capability for overall survival (OS) and disease-free survival (DFS), the paramount endpoints in evaluating prognostic tools. Patients were divided into high- and low-risk groups based on MER, AFP, tumor size, and BCLC. In the assessment of DFS, MER exhibited a remarkable ability to discriminate between low-risk and high-risk patients, with 5-year DFS rates of 78.3% and 17.6%, respectively (mean 4.1 years vs. 1.6 years, *p* < 0.001) (Figure 5a). Similarly, AFP identified a significant difference in DFS between low-risk and high-risk patients (72.2% vs. 38.1%, *p* = 0.018) with mean DFS of 4.0 years and 2.2 years, respectively. Conversely, tumor size did not show a significant differentiation in DFS between low-risk and high-risk patients (66.7% vs. 40.9%, *p* = 0.162) (Figure 5a). When evaluating OS, again, MER emerged as a significant predictor. The 5-year OS rates for MER low-risk patients and high-risk patients were 91.3% and 43.6% (mean OS of 4.7 years vs. 3.1 years, *p* = 0.002), respectively (Figure 5b). AFP and tumor size failed to prove as useful tools in predicting OS. Notably, BCLC staging, as it integrates patients’ performance, liver reserve, and tumor characteristics, proved to be significant in predicting both DFS and OS. These findings underscore the robust predictive capacity of MER in distinguishing between low-risk and high-risk patients for both DFS and OS in HCC patients undergoing liver resection.

Cox regression analysis revealed that MER (HR = 6.894; 95% CI 2.440–19.478; *p* < 0.001) and BCLC (HR = 5.041; 95% CI 1.901–13.370; *p* = 0.001) emerged as independent predictors for DFS, along with age (HR = 1.043; 95% CI 1.009–1.078; *p* = 0.013) and AFP (HR = 3.252; 95% CI 1.153–9.172; *p* = 0.026) (Figure 5c). However, when predicting OS, only BCLC (HR = 7.264; 95% CI 1.850–28.524; *p* = 0.004) and MER (HR = 8.152; 95% CI 1.707–38.925; *p* = 0.009) were identified as independent prognostic factors (Figure 5d). These findings underscore the remarkable promise of MER as a predictor, surpassing current clinicopathological factors and serum markers in its prognostic capabilities for both DFS and OS.

## 4. Discussion

Our study aimed to identify risk factors for predicting early recurrence in HCC patients after curative hepatectomy. Despite the common use of hepatectomy for its curative potential, the recurrence rate remains unacceptably high. Consequently, there is a crucial need to identify those at high risk of early recurrence to consider enhanced monitoring and adjuvant therapy as a potential solution. While previous studies relied on retrospectively collected data with diverse cancer stages and recurrence times, the markers discovered through this approach often had limited effectiveness due to overlapping recurrence times. To achieve more precise results, we employed a distinct approach by enrolling patients who experienced early recurrence within one year after hepatectomy, contrasting them with those who remained recurrence-free for five years. In our study, we conducted a comprehensive analysis of genome-wide methylation profiles to identify differential methylation changes between HCC patients with and without early recurrence. We delineated the distinct pathways involved and validated potential methylation markers and established a robust prediction model that accurately distinguishes early recurrence in HCC patients after hepatectomy. Notably, our methylation prediction outperforms commonly used clinicopathological predictors. Furthermore, by combining our model with other markers, we achieved robust prediction capabilities in identifying patients at risk of early recurrence.

Even after contemporary surgical resection with very low operative complications and mortality rate, the long-term survival of HCC patients remains disappointing because of the high incidence rate of recurrence. In particular, early recurrence is highly associated with worse post-recurrence survival and OS [7]. According to previous studies, the early recurrence rate may range from 41% to 49% [8,9]. Therefore, it is clinically required to identify patients at high risk of HCC early recurrence after hepatectomy. Until now, there are no well-recognized postoperative recurrence markers. Various factors associated with recurrence have been reported, including tumor-related factors, host liver-related factors, and even the type of surgery. Most studies were devoted to figuring out the correlation between recurrence after liver resection and clinicopathological characteristics. In the present study, we investigated the early recurrence of HCC from the viewpoint of global DNA methylation signatures and tried to identify valuable methylation markers for prognosis and prediction.

According to the gene ontology (GO) and pathway analysis, aberrant DNA methylation dramatically affects distinct gene signaling between early-recurrence patients with and without cirrhosis compared to recurrence-free patients. Cirrhosis-free patients with early recurrence exhibit impaired FGFR signaling, the PI3K network, the RUNX1/3-regulated pathway, the MAPK pathway, and tyrosine kinase receptor signaling, and elastic fiber formation and laminin interactions contributed to the epithelial–mesenchymal transition (EMT) in the progress of metastasis. The dysregulation of FGF/FGFR signaling is noted in many types of cancers, including HCC. FGFR2 and its corresponding ligands, FGF1 and FGF9, are associated with the EMT of HCC. The overexpression of FGF1 increased cell invasion ability and reduced apoptosis in HepG2 and SMMC-7721 cells [10]. FGFR2 was upregulated in poorly differentiated HCC and associated with portal vein invasion [11]. Moreover, highly expressed FGFR2 may potentially act as a predictor of HCC recurrence [12]. The PI3K/Akt signaling pathway is abnormally expressed in most malignancies and involves many essential functions in carcinogenesis, such as cell proliferation, apoptosis, cell invasion and metastasis, cell cycle regulation, angiogenesis, and the chemoresistance of chemotherapy. The versatile functions of the PI3K/Akt pathway make it an attractive target for anti-cancer therapy. RUNX3 is considered as a tumor suppressor gene, and its expression is significantly reduced in HCC tissues compared with that in adjacent normal tissues. Also, the hypermethylation of RUNX3 was observed in HCC [13]. Previous reports have described that RUNX3 functionally repressed the metastasis and invasion of HCC and increased E-cadherin expression [14].

On the other hand, in HCC patients with cirrhosis, early recurrence unveils aberrantly dominant pathways of HOX genes, FOXO-mediated transcription, p16INK4A-mediated CDK4 and CDK6 regulation, TP53, Wnt/beta-catenin, and Eph/ephrin signaling. HOX genes which control segment differentiation during embryonic development were first identified in Drosophila. Increasing studies indicate that the frequently anomalous expression of HOX genes is observed in various types of cancers, suggesting that these genes play a critical role in carcinogenesis. ZEB1 (zinc finger E-box binding Hox 1) and ZEB2 functioned as transcription factors, stimulating invasion and migration by enhancing EMT in cancer cells [15]. In HCC patients, ZEB1 expressions are associated with E-cadherin decreasing, venous invasion, and metastasis. Patients expressing high levels of ZEB1 and low levels of E-cadherin are prone to poorer prognosis [16]. FOXO1 is downregulated in HCC tumor tissues compared with normal liver tissues. A previous study demonstrated that FOXO1 suppresses invasion, metastasis, and EMT by the downregulation of ZEB2 through directly binding to the promoter region [17]. Moreover, ZEB2 overexpression is associated with HCC recurrence [18]. The activation of Wnt/beta-catenin signaling is very important in HCC development and corresponds to our results existing in hypomethylated gene clustering. A previous study also demonstrated that Wnt/beta-catenin plays a pivotal role in HCC early recurrence [19]. EPH/ephrin involves cell migration, axon guidance, and synapse formation during embryonic development and also participates in carcinogenesis through cell adhesion, motility, and cell–matrix interactions. The elevation of EPH/ephrin expression is correlated with recurrence in various cancers, including HCC [20].

Crucial hub genes regulated by DNA methylation and involved in early recurrence after resection were identified by the STRING database. In cirrhotic patients, EGF, CDH1, and FGF13 were identified to play important roles in early recurrence. A large proportion of HCC cases can be found in viral infection or liver damage caused by alcohol consumption. This ongoing damage leads to inflammation and further cirrhosis in the liver. EGF and its receptor (EGFR) may act as a bridge between inflammation and liver cancer. EGF activates the expression of CXCL5 and CXCL8, a kind of inflammatory factor, in HCC cells, contributing to increasing the metastatic ability of HCC cells [21]. In addition, several studies have demonstrated that EGF/EGFR overexpression results in aggressive HCC and metastasis by activating AKT/ERK signaling to regulate MMP2, disrupting desmosomes and adherens junctions, as well as decreasing E-cadherin, encoded by CDH1 [22,23].

In non-cirrhotic patients, we found that BDNF is a pivotal hub gene involved in early recurrence. BDNF (brain-derived neurotrophic factor) belongs to the group of neurotrophins which participate in neurogenesis and brain development. Over the past decades, BDNF also has been found to take part in cancer development, including brain, breast, urinary, gastric, colon, pancreas, and liver cancer. BDNF binding to the TrkB receptor triggers the MAPK, PI3K, EGFR, and PLC-g (phospholipase C-gamma) pathways and may contribute to its oncogenesis and metastasis [24,25]. A previous study indicated that the overexpression of BDNF and TrkB was observed in HCC specimens, and BDNF-neutralizing antibody or Trk inhibitor K252a effectively induced apoptosis and suppressed the invasion of HCC cell lines [26]. Our study is the first to report that BDNF hypomethylation frequently presents in HCC patients and plays an important role in early recurrence development.

Also, our study is the first one to unveil that HCC early recurrence is correlated with the hypermethylation of LTB4R2 and hypomethylation of MEIS3, FOXL2, PLA2G7, LMO7, BDNF, and NCAM1. LTB4R2 has been reported to be one of the 33-IRGP (immune-related gene pair) prognostic signature of HCC [27]. Elevated MEIS3 exerts HCC cell viability, migration, and invasion through HOXA1, directly binding to its enhancer [28]. A recent study showed that PLA2G7 highly expressed in HCC tumor tissue compared with normal tissue may act as a diagnostic marker of HCC [29]. NCAM1 is highly expressed in hepatic stem cells and is related to the function of EMT. Combining with these novel methylation markers associated with HCC early recurrence, we successfully established a methylation prediction model to distinguish ER from RF with superior performance compared with some current predictors, including tumor size, AFP, and BCLC. Furthermore, MER may act as an independent prognostic factor for the assessment of OS and DFS.

Among the various predictors examined in our study, MER plays a crucial role in predicting DFS and OS. While BCLC also demonstrates notable predictive power for DFS and OS, it is essential to note that BCLC is a composite predictor, encompassing tumor characteristics, host performance, and liver reserve. On the other hand, MER, despite being solely a gene-related predictor, still exhibits significant power in predicting both DFS and OS. Cox regression analysis revealed that only MER and BCLC were identified as independent prognostic factors. These compelling findings underscore the remarkable promise of MER as a predictor, surpassing current clinicopathological factors and serum markers in its prognostic capabilities for both DFS and OS.

In patients undergoing liver resection, recurrence is a significant clinical challenge. Patients with early recurrence have a shorter cancer-free survival (8.4 months) compared to a median of 21.3 months for patients with late recurrence [30], underscoring the critical importance of robust prognostic assessment. For high-risk patients with early recurrence, the early diagnosis of recurrence increases the likelihood of eligibility for curative treatments, such as radiofrequency ablation (RFA), thereby potentially improving survival rates [31]. Given the high recurrence rate, several studies have demonstrated the efficacy of antiviral therapy, such as interferon treatment, in preventing recurrence after curative treatment for hepatitis virus-related HCC [32]. Beyond hepatitis virus infection, metabolic dysfunction-associated fatty liver disease (MAFLD) and obesity are also established risk factors for HCC development. Furthermore, recent studies have shown an association between diabetes mellitus (DM) and a higher incidence of HCC. Several pharmacological agents have shown promise in managing HCC in patients with diabetes, including antidiabetic medications, antineoplastic agents, and other therapies such as statins and aspirin [33]. These findings highlight the importance of prognostic values after liver resection to predict high-risk patients for early recurrence. Adjuvant therapy may benefit high-risk patients by reducing recurrence and prolonging survival.

While the study’s pilot extrapolatory nature allowed for the intentional enrollment of patients experiencing early recurrence (ER) within one year and those remaining recurrence-free (RF) for five years after liver resection, it is important to recognize several limitations. First, the study design follows a retrospective approach, potentially introducing biases and influencing comprehensive variable capture. Secondly, the limited sample size further restricts the study’s ability to draw comprehensive conclusions or extrapolate findings to broader populations. Despite these considerations, the study’s valuable findings highlight new methylation patterns associated with early recurrence and underscore the superior predictive power of the methylation-based model (MER) in RFS and OS.

## 5. Conclusions

Our study demonstrates the role of aberrant DNA methylation in early recurrence among HCC patients undergoing curative resection. This dysregulation affects key signaling pathways, with newly identified genes that might serve as potential methylation markers. Furthermore, we established a methylation-based prediction model for distinguishing ER form RF, the performance of which is superior to current clinical predictors. Given the high incidence of early recurrence after curative hepatectomy, effective and precise predictors of ER are essential for vigilant monitoring, formulating proper adjuvant strategies and ultimately improving patients’ outcomes.

## Figures and Tables

**Table 1:** Clinical characteristics of HCC patients.

Characteristics		Testing, *n* = 24 (%)	Validation, *n* = 40 (%)
Age (median)		59	56
Gender			
	Male	20 (83.3)	28 (70)
	Female	4 (16.7)	12 (30)
Hepatitis			
	HBV−/HCV−	4 (16.7)	10 (25)
	HBV+	17 (70.8)	20 (50)
	HCV+	2 (0.08)	8 (20)
	HBV+/HCV+	1(0.04)	2 (5)
AFP value (ng/mL) (median)		13	24
BCLC staging			
	0-A	12 (70.6)	26 (66.8)
	B-C	5 (29.4)	13 (33.2)
pTNM staging			
	Stage 1	9 (47.4)	15 (42.9)
	Stage 2	4 (21)	15 (42.9)
	Stage 3	2 (31.5)	5 (14.2)
Diameter of largest HCC nodule			
	<5 cm	19 (79.2)	21 (52.5)
	≥5 cm	5 (20.8)	19 (47.5)
Histologic grade			
	Well	4 (23.5)	5 (12.8)
	Moderately	10 (58.8)	27 (69.2)
	Poorly	3 (17.6)	7 (17.9)
Status of non-tumor liver			
	Non-cirrhosis	14 (58.3)	40 (100)
	Cirrhosis	10 (41.7)	0 (0)
Early recurrence			
	Yes (ER group)	11 (45.8)	20 (50)
	No (RF group)	13 (54.2)	20 (50)

**Table 2:** Sensitivity and specificity of predictors for early recurrence.

	**AUC (95% CI)**	**Sensitivity (95% CI)**	**Specificity (95% CI)**	***p*-Value**
Tumor Size	0.754 (0.600–0.909)	73.7 (64.1–83.3)	57.1 (45.8–68.4)	0.006
BCLC	0.772 (0.619–0.926)	61.1 (40.2–81.9)	90.5 (76.9–100)	0.004
AFP	0.683 (0.511–0.854)	72.7 (53.6–91.7)	61.9 (39.4–84.3)	0.052
MER	0.855 (0.738–0.971)	85.7 (70.7–100)	73.7 (53.9–93.4)	<0.001
MER + AFP	0.868 (0.756–0.979)	85.7 (70.7–100)	72.2 (51.5–92.8)	<0.001
AFP + Size	0.767 (0.616–0.919)	85.7 (70.7–100)	66.7 (44.9–88.4)	0.004
AFP + BCLC	0.739 (0.569–0.910)	85.7 (70.7–100)	73.7 (52.7–94.6)	0.012
Size + BCLC	0.780 (0.629–0.932)	85.7 (70.7–100)	66.7 (44.9–88.4)	0.003
MER + Size	0.920 (0.838–1.000)	81.0 (64.2–97.7)	89.5 (76.0–100))	<0.001
MER + BCLC	0.931 (0.857–1.000)	81.0 (64.2–97.7)	94.4 (84.3–100)	<0.001
MER + AFP + BCLC	0.944 (0.662–0.951)	85.7 (70.7–100)	82.4 (64.2–100)	<0.001
MER + AFP + Size + BCLC	0.952 (0.893–1.000)	90.5 (77.9–100)	88.2 (72.8–100)	<0.001

## Data Availability

The datasets used and analyzed in the current study are available from the corresponding author upon reasonable request.

## Abbreviations

The following abbreviations are used in this manuscript:

AFP	alpha-fetoprotein
AUC	area under the curve
BCLC	Barcelona clinic liver cancer classification
COBRA	combined bisulfite restriction analysis
ER	early recurrence
FGFR	fibroblast growth factor receptors
HCC	hepatocellular carcinoma
MAPK	mitogen-activated protein kinase
MER	methylation prediction model for ER
PI3K	phosphoinositide 3-kinases
qMSP	real-time quantitative methylation-specific PCR
RF	recurrence-free
ROC	receiver operating characteristic curve
